# Clinically relevant investigation of flattening filter‐free skin dose

**DOI:** 10.1120/jacmp.v17i6.6307

**Published:** 2016-11-08

**Authors:** Christopher L. Guy, Kishor Karki, Manju Sharma, Siyong Kim

**Affiliations:** ^1^ Department of Radiation Oncology Virginia Commonwealth University Richmond VA; ^2^ Department of Radiation Oncology University of Rochester Medical Center Rochester NY USA

**Keywords:** flattening filter‐free, FFF, skin dose, field size, beam energy, multileaf collimator

## Abstract

As flattening filter‐free (FFF) photon beams become readily available for treatment delivery in techniques such as SBRT, thorough investigation of skin dose from FFF photon beams is necessary under clinically relevant conditions. Using a parallel‐plate PTW Markus chamber placed in a custom water‐equivalent phantom, surface‐dose measurements were taken at 2×2,3×3,4×4,6×6,8×8,10×10,20×20, and $30\times 30\;{\text{cm}}^{2}$ field sizes, at 80, 90, and 100 cm source‐to‐surface distances (SSDs), and with fields defined by jaws and multileaf collimator (MLC) using multiple beam energies (6X, 6XFFF, 10X, and 10XFFF). The same set of measurements was repeated with the chamber at a reference depth of 10 cm. Each surface measurement was normalized by its corresponding reference depth measurement for analysis. The FFF surface doses at 100 cm SSD were higher than flattened surface doses by 45% at $2\times 2\;{\text{cm}}^{2}$ to 13% at $20\times 20\;{\text{cm}}^{2}$ for 6 MV energy. These surface dose differences varied to a greater degree as energy increased, ranging from +63% at $2\times 2\;{\text{cm}}^{2}$ to −2% at $20\times 20\;{\text{cm}}^{2}$ for 10 MV. At small field sizes, higher energy increased FFF surface dose relative to flattened surface dose; while at larger field sizes, relative FFF surface dose was higher for lower energies. At both energies investigated, decreasing SSD caused a decrease in the ratios of FFF‐to‐flattened surface dose. Variability with SSD of FFF‐to‐flattened surface dose differences increased with field size and ranged from 0% to 6%. The field size at which FFF and flattened beams gave the same skin dose increased with decreasing beam energy. Surface dose was higher with MLC fields compared to jaw fields under most conditions, with the difference reaching its maximum at a field size between $4\times 4\;{\text{cm}}^{2}$ and $6\times 6\;{\text{cm}}^{2}$ for a given energy and SSD. This study conveyed the magnitude of surface dose in a clinically meaningful manner by reporting results normalized to 10 cm depth dose instead of depth of dose maximum.

PACS number(s): 87.53.Bn, 87.53.Ly, 87.55.‐x, 87.55.N‐, 87.56.N‐

## I. INTRODUCTION

The removal of the flattening filter in conventional medical linear accelerators produces a beam with decreased average energy, since lower‐energy photons are no longer attenuated. In turn, the unattenuated beam increases monitor unit efficiency. The primary contributor of head scatter reaching the patient or phantom in flattened beams is mainly due to the flattening filter; therefore, removal of the filter greatly decreases head scatter which can lead to decreased out‐of‐field dose.^(1)^ Perhaps the greatest benefit of beams without the flattening filter in place, herein referred to as FFF beams, is the large increase in dose rate, up to four times that of flattened beams. The high dose rates achievable with unflattened beams greatly reduce treatment times, minimizing intrafraction motion and, in some cases, enabling beneficial techniques such as breath‐hold or active breathing control to be used for otherwise ineligible patients at our institution.

Early investigations into the dosimetric properties of FFF beams were performed on modified linacs with the flattening filter manually moved out of the beamline.^2^, ^3^ Linacs with FFF beams have since been released by vendors and implemented clinically at many institutions. Properties of the FFF beams for these machines, usually determined during the commissioning process, have been subsequently reported.^4^, ^5^, ^6^ Comparisons between Monte Carlo calculations and experimental measurements have been performed to confirm that simulations accurately model accelerator geometry.^7^, ^8^, ^9^

While much has been reported on depth‐dose properties of unflattened beams, limited data on surface dose of these beams were reported by the previously mentioned investigations. A study by Wang et al.^(10)^ specifically focused on the impact of flattening filter removal on superficial dose by measuring ionization curves in the buildup region with various field sizes made using jaws and multileaf collimators (MLCs) together. However, only a single source‐to‐surface distance (SSD) was used, and reported measurements were normalized by the same maximum depth dose of a $10\times 10\;{\text{cm}}^{2}$ field size. The goal of this investigation is to measure skin dose of FFF beams under clinically relevant conditions. In this study, we report skin dose measurements over a large range of field sizes at multiple SSDs using both fields made with collimator jaws and those made using the MLC alone.

## II. MATERIALS AND METHODS

### A. Photon beams

Measurements were made using a Varian TrueBeam linear accelerator (Varian Medical Systems, Palo Alto, CA) which was commissioned in June 2013 for flattened photon beams and has since been used for clinical treatments. In October 2014, unflattened beams of energies 6 MV and 10 MV were commissioned. The maximum dose rates were 600, 600, 1400, and 2400 MU/min for the 6 MV flattened, 10 MV flattened, 6 MV FFF, and 10 MV FFF beams, respectively. For all measurements, 500 MU were delivered to minimize statistical uncertainty, and both the gantry and collimator angles were set at 0°.

### B. Equipment

**Figure 1:**
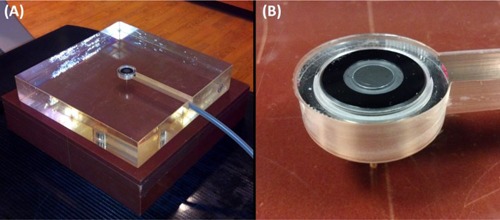
Experimental measurement setup: (a) experimental setup is shown for surface measurements using the water‐equivalent phantom; (b) close‐up view of the Markus chamber set in the custom‐machined phantom for surface measurements.

Skin dose was measured using a Markus parallel‐plate ionization chamber (PTW Type 23343, PTW, Freiburg, Germany) which has a nominal sensitive volume of $0.055\;{\text{cm}}^{3}$ (2.65 mm radius, 2 mm depth) and an entrance foil thickness of 0.03 mm (polyethylene, $2.76\;\text{mg}/{\text{cm}}^{2}$ density). The chamber was embedded in a custom‐machined $25\times 25\times 5\;{\text{cm}}^{3}$ Rexolite 1422 (cross‐linked polystyrene, density $1.05\;g/{\text{cm}}^{3}$) phantom which was placed on top of solid water slabs of 10 cm thickness for backscatter, as shown in Fig. 1. Reference depth dose was obtained by placing an additional 10 cm thick solid water slab on the setup of Fig. 1. Measurements were made with a Keithley 35614 electrometer.

### C. Measurements

For each field size, SSD, and energy, four measurements were taken: two at +300 V bias and two at −300 V bias. These four measurements were averaged together to account for polarity bias of the chamber. As measurements were taken over several days, output measurements were made at the beginning of each session using the $10\times 10\;{\text{cm}}^{2}$ jaw field size at 100 cm SSD and taken for each energy to correct for the daily variations in beam output.

**Figure 2:**
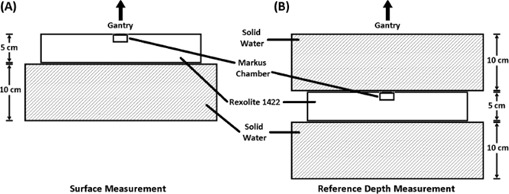
Diagrams of measurement geometries are shown for (a) surface measurements and (b) reference (10 cm) depth measurements. The appropriate SSD was maintained when switching between the two geometries.

To investigate the effect of field size on surface dose, field sizes of 2×2,3×3,4×4,6×6,8×8,10×10,20×20, and $30\times 30\;{\text{cm}}^{2}$ were first made using only the collimator jaws. The jaws were then set to $10\times 10\;{\text{cm}}^{2}$, and the MLC was used to create the square fields of 2×2,3×3,4×4,6×6, and $8\times 8\;{\text{cm}}^{2}$. This method of using MLCs to create fields more closely mimics the clinical scenario during stereotactic body radiotherapy (SBRT) treatments based on IMRT, where the jaws can be set to the maximum field size of all segments while the MLC leaves produce the smaller, complex shapes. A complete set of measurements were made at three SSDs: 80, 90, and 100 cm. All surface measurements were repeated for normalization purposes with the chamber at a depth of 10 cm ($d_{10}$), achieved by adding 10 cm thick solid water on top of the detector. Diagrams of measurement geometries for surface and reference depth are shown in Fig. 2.

### D. Dose ratios

Absolute surface dosimetry is difficult; therefore, relative surface dose measurements were made by normalizing surface values to those at $d_{10}$ for each field size (i.e., $D_{\text{surf}}/D_{d10}$) and will herein be referred to simply as surface dose. A depth of $d_{10}$ was chosen rather than depth of dose maximum ($d_{\max}$) since 10 cm depth is closer to the clinically meaningful prescription point.

Markus chambers are widely used for surface measurements, but they require correction factors, which are functions of beam quality, to account for overresponse. Correction factors have previously been obtained through extrapolation chamber measurements,^(11)^ but were unavailable at our institution. Instead, the effect of chamber response was minimized by taking the ratio of surface doses (e.g., ${\left(D_{\text{surf}}/D_{d10}\right)}^{\text{FFF}}/{\left(D_{\text{surf}}/D_{d10}\right)}^{\text{flattened}}$, enabling investigation into the effects of collimation type, field size, SSD, and the flattening filter.

## III. RESULTS

As measurements were performed over several weeks, separate surface measurements were performed during each session to account for daily variability in beam output and were obtained for all energies using a $10\times 10\;{\text{cm}}^{2}$ jaw field. The output‐corrected values were used in the analyses of the following subsections.

### A. Flattened versus FFF

**Figure 3:**
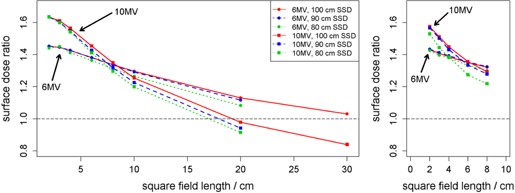
Ratios of FFF‐to‐flattened surface dose are shown as a function of field size in water‐equivalent phantom for jaw fields (left) and MLC fields (right). For MLC fields, jaws were set to $10\times 10\;{\text{cm}}^{2}$. Each surface measurement was first normalized by the reference $d_{10}$ measurement of the same field size. The same legend applies to both subfigures.

Measurements were taken under all setup conditions for both flattened and FFF beams. The ratios of FFF to flattened surface dose are shown in Fig. 3. A decrease in the ratio was observed with both increasing field size and decreasing SSD. 6 MV surface dose ratios showed less variation than 10 MV ratios across the range of field sizes investigated. For small jaw field sizes, SSD had little effect on the ratio; however, as field size was increased, the ratios at the differing SSDs showed greater difference. For example, at $2\times 2\;{\text{cm}}^{2}$ jaw field size, surface dose ratios differed from 80 cm SSD to 100 cm SSD by 0.8% and 0.0% for 6 MV and 10 MV, respectively. However, at $20\times 20\;{\text{cm}}^{2}$ field size, these differences in ratios increased to 4.5% and 6.9%. Similar trends were observed for MLC fields.

At the smallest field sizes ($\leq 3\times 3\;{\text{cm}}^{2}$), surface dose of FFF beams was about 63% greater than flattened beams at 10 MV and approximately 45% greater at 6 MV using jaw fields. The increase in surface dose at small field sizes for FFF beams was slightly reduced when using MLC fields to about 55% and 43% for 10 MV and 6 MV, respectively.

### B. MLC versus jaw fields

**Figure 4:**
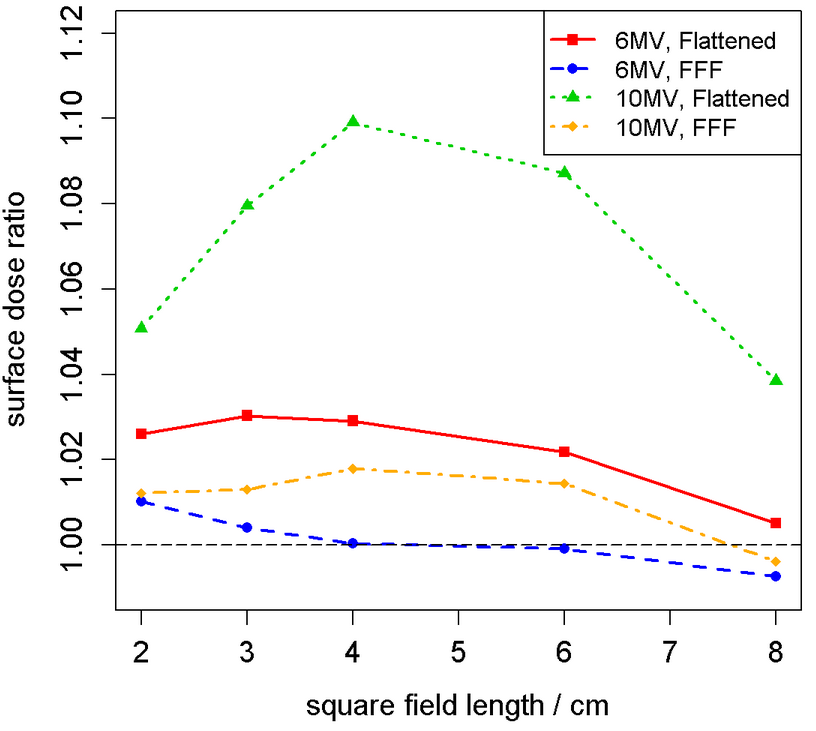
Ratios of MLC‐to‐jaw field surface dose in water‐equivalent phantom at each energy as a function of field size at 100 cm SSD. For MLC fields, jaws were set to $10\times 10\;{\text{cm}}^{2}$. Each surface measurement was first normalized by the reference $d_{10}$ measurement of the same field size.

The ratios of surface dose with MLC fields to surface dose with jaw fields are shown in Fig. 4. A peak in observed difference in dose between MLC and jaw fields was seen at $4\times 4\;{\text{cm}}^{2}$ field size for 10 MV beams. At all SSDs, the ratio between MLC and jaw field measurements was relatively stable across field sizes for FFF beams (average arithmetic range in ratios across field size of 0.019, taken across all SSDs and energies); whereas larger variability was seen for flattened beams (average arithmetic range in ratios across field size of 0.045).

### C. Reference depth‐dose comparison

**Figure 5:**
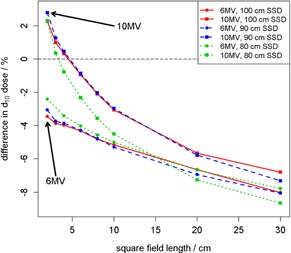
FFF‐to‐flattened depth‐dose differences. Percentage differences between FFF and flattened doses at a reference depth of 10 cm.

The differences in dose at $d_{10}$ between FFF and flattened beams are shown in Fig. 5. Because no beam tuning to match FFF beams with their corresponding flattened beams was made for the TrueBeam, the dose difference at $d_{10}$ is expected even for the $10\times 10\;{\text{cm}}^{2}$ reference field. With increasing field size, flattened dose became increasingly larger (by about 8% at $30\times 30\;{\text{cm}}^{2}$). As field size increased, the scatter contribution to reference depth dose increased as well. However due to the flatness of the dose profile across the field, flattened dose varied strongly with field size, explaining the observed difference in $d_{10}$ dose. This effect was present in the surface dose results of Figs. 3 and 4.

### D. Uncertainty analysis

Uncertainty of the reported ratios was calculated via measurement error propagation. The electrometer reported collected charge with 1 pC precision. Based on the variance of repeat measurements for each data point, the standard deviation of the FFF‐to‐flattened ratios for 6 MV beam energy at 100 cm SSD was 0.0008 on average across field sizes from $2\times 2\;{\text{cm}}^{2}$ to $20\times 20\;{\text{cm}}^{2}$ and ranged from 0.0003 to 0.0016. This uncertainty was relatively small compared to the magnitude of the ratios (<0.2%). The variability at other energies and SSDs and for MLC‐to‐jaw ratios was of similar magnitude.

## IV. DISCUSSION

Removal of the flattening filter affects surface dose in numerous ways. The decrease in treatment head scatter of the photon portion of the incident beam, as described in the Introduction, leads to a reduction in dose at all depths. More important for surface dose is the change in electron composition of the beam, which is the primary contributor of skin dose. A number of photons generated in the target interact with the target itself to produce secondary electrons which can travel downstream.^(12)^ Accelerators built with flattening filter‐free capabilities, such as the Varian TrueBeam, have filters above the monitor chambers to remove electron contamination. However, contaminant electrons are still allowed to reach the patient.

With external beam radiotherapy, surface dose is essentially a function of prescription dose. By prescribing to shallower depths, the surface dose decreases. Studies often report surface dose as a percentage of that at $d_{\max}$ which is much greater and therefore may bias one into thinking surface dose is relatively low. Yet tumors and their prescription points are often located at depths well beyond $d_{\max}$. Thus, it is more clinically useful to use $d_{10}$ as the reference depth for surface dose reporting, as has been done in this study.

Field size determines the surface dose of a given beam, as well. Flattened and unflattened beams are affected differently by both field size and the attenuation devices used to create the fields. With the flattening filter in place, differential attenuation of both photons and contaminant electrons occurs across the width of the field, with less attenuation occurring peripherally. It is expected, and observed in Fig. 4, that flattening filter‐free beams show little variation with field size since equal filtration occurs across the entire field width.

When MLC leaves are in the path of the beam, photons of the primary beam interact with the MLC material and generate scattered photons and secondary electrons. Electrons of this nature which reach the surface mostly originate along the inner vertical edges of the collimator and have angular distributions which increase with energy. The additional electrons cause an elevation in surface dose. A peak in surface dose ratio of MLC‐to‐jaw fields is evident for the 10 MV FFF beam in Fig. 4 at a field size of about $4\times 4\;{\text{cm}}^{2}$. We suspect that this peak is due to the trade‐off between the number of electrons being produced in the MLC leaves and the distance of the MLC leaves to the point of measurement. However, this supposition requires future Monte Carlo simulation for validation.

Clinical use of unflattened photon beams in radiotherapy holds most promise with hypofractionated SBRT treatments where large doses are delivered in a small number of fractions. If flattened and FFF beams are calibrated identically (e.g., to deliver 1 cGy at reference depth per monitor unit), then the use of FFF beams will increase efficiency by shortening treatment time. On a TrueBeam, an unflattened beam can be delivered at a dose rate four times larger than the flattened counterpart at 10 MV (more than twice as large at 6 MV), which would enable beam‐on time reductions of one half to one quarter. This increase in treatment efficiency only applies to beam‐on time, so longer treatments such as SBRT cases would see the maximum benefit in using unflattened beams. SBRT commonly utilizes either arcs or multiple beams to increase dose falloff. However, this does not necessarily guarantee that there will be no skin complication. In fact, skin complication can be severe in SBRT because of its hypofractionated nature resulting in high‐fractional dose. Serious skin complications have been reported with SBRT.^(13)^ In addition, from time to time, the desire of having a greater number of beams is compromised by the necessity of beam path avoidance for critical structures, resulting in increased skin dose in SBRT.

In principle, it may be possible to extract skin doses from commissioning data. However, depending on the situation, it may be difficult to accomplish in reality. The most common method of skin dose measurement in actual practice is to use a parallel‐plate chamber with the necessary corrections applied as described in this study, which is the next best option after expensive and exhausting extrapolation chamber measurement. However, to the best of our knowledge, many users utilize a cylindrical chamber for beam scanning during commissioning. And it is well‐known that cylindrical chambers suffer from significant volume averaging effect in high‐gradient regions (e.g., buildup regions) which is worst at the surface. Thus, skin dose estimation from cylindrical chamber data is not reliable, and we therefore believe it is clinically useful to have skin dose data publicly available.

## V. CONCLUSIONS

This study has investigated the ratios between skin doses with and without a flattening filter under conditions which are pertinent to modern treatment techniques involving beam modulation. Surface measurements were evaluated as a function of both jaw‐defined and MLC‐defined field sizes and for multiple SSDs. From these data, the following conclusions can be drawn:

1. As SSD increases, skin dose decreases relative to dose at depth.
2. The difference in skin dose between MLC and jaw fields is less variable with field size for FFF beams compared to flattened beams.
3. With increasing beam energy, field size has a greater effect on the difference in skin dose between FFF and flattened beams.
4. Skin dose is higher with FFF beams at small field sizes than with flattened beams, while flattened beams give higher skin dose at large field sizes.
5. The field size at which FFF and flattened beams give the same skin dose increases with decreasing beam energy.

To the best of the authors’ knowledge, this is the first study to evaluate surface dose as a function of (10 cm) depth dose which is more closely related to the treatment prescription point than $d_{\max}$, enhancing clinical relevance.

## COPYRIGHT

This work is licensed under a Creative Commons Attribution 3.0 Unported License.

## Supporting information

Supplementary Material

Supplementary Material

Supplementary Material
