# Supplementary figures and images for: Clinically relevant investigation of flattening filter‐free skin dose
Source: J Appl Clin Med Phys. 2016 Nov 8;17(6):140–8. doi: 10.1120/jacmp.v17i6.6307 (PMC5690509; doi:10.1120/jacmp.v17i6.6307)

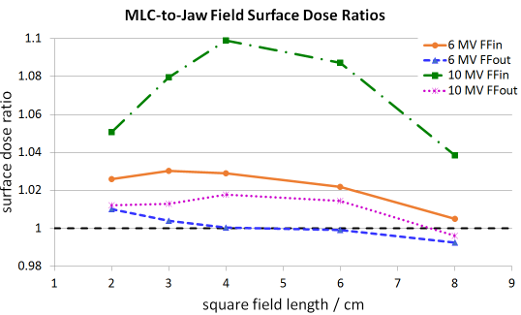

Supplement: Supplementary file 1 — Supplementary Material [file ACM2-17-140-s001.png]

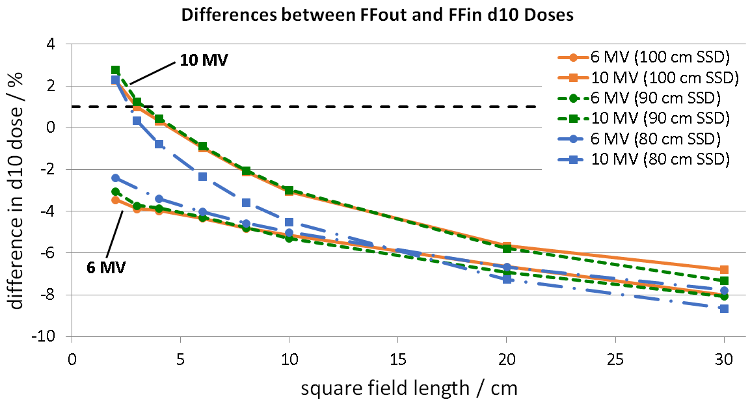

Supplement: Supplementary file 2 — Supplementary Material [file ACM2-17-140-s002.png]
