# Supplementary material for: Clinically relevant investigation of flattening filter‐free skin dose
Source: J Appl Clin Med Phys. 2016 Nov 8;17(6):140–8. doi: 10.1120/jacmp.v17i6.6307 (PMC5690509; doi:10.1120/jacmp.v17i6.6307)
Supplement: Supplementary file 3 — Supplementary Material [file ACM2-17-140-s003.rtf]

Reviewer A:

This is an improved version of this manuscript. I have concern about the
content in the first paragraph of the discussion. Otherwise, I find this
work to be reasonable.

Ln 165 “The thin target in the treatment head allows some portion of the
incident primary electron beam to penetrate through and travel to the
patient.”

I do not believe this is correct for clinical FFF beams. This phenomenon was
observed when the flattening filter was completely removed and there was no
beam filtration (Titt 2006). This was rectified by placing 0.8 mm of Brass
in place of the flattening filter (see Xiao 2015 as previously suggested) in
clinical Varian FFF beams. This thin plate of metal removes these electrons.

Ln 169 (and Ln 173). “This allowed for the saturation of monitor unit
chambers due to electron contamination which limited the number of electrons
reaching the patient (12)”

I can not see which reference this is because the reference has been
redacted (for future submissions and even future versions of this work,
references such as this should not be redacted for submission to JACMP
because it does not indicate who the authors of the current manuscript are.
Therefore there is no conflict with including the reference information,
which should be done so the reviewers can see what references are being
cited). However, to the point, I am not aware of any papers that caution
saturation of the monitor unit chamber due to electron contamination.
Indeed, Titt 2006 studies explicitly the signal in the monitor chamber from
electrons, and saturation is never mentioned.

This discussion of sources and influences of surface dose should be revised.
Response:
The first paragraph of the discussion (beginning on line 161 of the revised manuscript) was changed to address the concerns of the reviewer. Also, all references were included which do not indicate the authors of the current work.
